# The high platelet counts as predictor for early foetal demise

**DOI:** 10.1080/07853890.2021.1968027

**Published:** 2021-08-25

**Authors:** Xiaowen Shao, Dandan Wang, Yue Xu, Ling Guo, Hui Yang, Jieru Zhou, Jiayi Liang, Jie Qian, Jiajing Cheng, Lihua Sun, Yaozu Xiang

**Affiliations:** a Department of Obstetrics and Gynecology, Tongji University School of Medicine, Shanghai Tenth People’s Hospital, Shanghai, China; b School of Life Sciences and Technology, Tongji University, Shanghai, China; c Department of Reproductive Medicine Center, Tongji University School of Medicine, Shanghai East Hospital, Shanghai, China; d Department of Health Management, Tongji University School of Medicine, Shanghai East Hospital, Shanghai, China; e School of Mathematics and Statistics, University of Glasgow, Glasgow, UK

**Keywords:** Pregnancy, early foetal demise, platelet counts, mean platelet volume

## Abstract

**Objectives:**

Early fetal demise (absence of cardiac activity in a visible fetus) is a very common event, but there are no reliable biomarkers to predict it. The purpose of the study was to assess the association of platelet parameters with early fetal demise.

**Methods:**

In this case-control study, we included women with normal deliveries or those ultrasound diagnosed as early fetal demise. For those who were identified with early fetal demise, the platelet parameters were analyzed before the ultrasound diagnosis, which is based on the absence of either an embryo within a gestational sac or cardiac activity in a visible embryo in the 5-10 weeks of gestation. The association between the risk of early fetal demise with the women's mean platelet volume (MPV) and platelet counts was calculated by logistic regression. Duplicate measurements of platelet aggregation were performed with VerifyNow.

**Results:**

In total, 99 women identified with early fetal demise and 170 women who had an uncomplicated pregnancy with normal delivery from January 2017 and August 2020 were included in the study. We found that platelet counts in the early fetal demise group were significantly higher than healthy pregnancies. In addition, platelet reactivity is higher in the normal delivery group than those in early fetal demise group (*p* < .05). High levels of platelet counts resulted in an adjusted odds ratio (OR) of 2.075 (95% confidence interval [95% CI], 1.215–3.544; *p* = .008) for early fetal demise.

**Conclusions:**

Increased platelet counts in the first trimester may be a predictor for the risk of early fetal demise.

## Introduction

Platelets, produced from megakaryocytes mainly in the bone marrow and lung, are implicated not only in haemostasis and thrombosis [1], but also in other physiological and pathophysiological processes [2,3]. Mean platelet volume (MPV), a measure of platelet size, is a potential biological marker of platelet function [4] and is commonly available in the clinical practice. Platelets with increased MPV are more active and prothrombotic and are thus more prone to platelet adhesion and aggregation [4–7]. Higher MPV was shown to be associated with several cardiovascular risk factors such as diabetes and hypertension [4,8] and is associated with an increased risk for both arterial and venous thrombosis [9,10]. Patients with pre-existing coronary artery disease and increased MPV are at higher risk of myocardial infarction [11]. Although MPV is inversely correlated with platelet counts in healthy individuals [12,13], the levels of and correlation between MPV and platelet counts have not been determined during pregnancy, especially in early foetal demise (absence of cardiac activity in a visible embryo). More importantly, there is no current report linking MPV and platelet counts to healthy pregnancy or early foetal demise.

Miscarriage or early pregnancy loss is the most common complication of pregnancy with a reported rate of 12–24% [14]. Early foetal demise is one of the most severe types of miscarriage [15]. Early foetal demise occurs in 10% of all clinically recognized pregnancies and 80% of pregnancy losses occur in the first trimester [16]. Early foetal demise impacts around one million women in the United States each year [17]. Although genetic abnormalities are the most common cause of first trimester miscarriage, many other factors including the prothrombotic state may also contribute [14]. Interestingly, recent report shows that starting from the first trimester, platelet counts decrease in all pregnant women [18]. Consistently, it has been long recognized that pregnancy induces hypercoagulability, a physiologic change that prevents postpartum haemorrhage. The clinical importance of platelets in regulating haemostasis is highlighted by the bleeding and thrombotic complications. However, it remains unknown whether platelet counts could influence and reflect the survival of the foetus during early stage of pregnancy. We speculate that abnormal MPV alone or together with changed platelet counts are associated with early foetal demise.

Many pregnancy-related complications and pre-existing disorders may cause thrombocytopenia or increased platelet counts. Multiple physiological changes including increased plasma volume [19], spleen size [20] and placental circulation [21] during pregnancy, also contribute to lower platelet counts. Since higher MPV are found in menstruating women and women taking oral contraceptives, or women with white blood cell counts, fibrinogen concentrations and platelet [8,22], we excluded those with pregnancy-related complications or pre-existing disorders associated with the change of platelet counts. In the present case-control study, we ascertained the MPV and platelet counts in pregnant women with healthy deliveries or those diagnosed as early foetal demise in the Shanghai Tenth People’s Hospital between 2017 and 2020. We aim to evaluate whether abnormal platelet counts confer an increased risk for early foetal demise.

## Methods

### Study design and participants

We collected and analysed the platelet parameters in women (median age, 30) at multiple time-points during pregnancy. Among 1284 pregnant women, 596 were diagnosed with early foetal demise (median age, 32) in the Shanghai Tenth People’s Hospital between 2017 and 2020. Demographic data, MPV, platelet counts, medical-record numbers and diagnosis codes identified from electronic medical records were combined into a single database. To focus a direct association between platelet counts, MPV and early foetal demise, we excluded women with pregnancy-related complications or pre-existing disorders that were considered to be associated with thrombocytopenia. Recorded pregnancy-related complications were hypertension, diabetes before or during pregnancy, preeclampsia, HELLP (haemolysis, elevated liver enzymes and low platelet counts) syndrome, preterm birth (defined as birth before 37 weeks’ gestation), stillbirth and placental abnormalities (e.g. placental abruption, placenta previa or placenta accreta). Pre-existing disorders that were considered to be associated with thrombocytopenia were recorded as immune thrombocytopenic purpura, systemic lupus erythematosus, human immunodeficiency virus infection and hepatitis B or C.

We included 23 normal pregnant women and 50 patients with early foetal demise. Duplicate measurements of platelet aggregation were performed with VerifyNow® Aspirin in normal pregnant women in 5–10 weeks’ gestation, and in patients with early foetal demise when ultrasound diagnosis confirmed.

Our study protocol was approved by the ethical committees of Shanghai Tenth People’s Hospital (No: SHSY-IEC-4.1/19-11/01) and Shanghai East Hospital.

### Early foetal demise

Inclusion criteria: The ultrasound diagnosis of early foetal demise is based on the absence of either an embryo within a gestational sac or cardiac activity in a visible embryo in 5–10 weeks’ gestation.

### Platelet counts and MPV

Venous blood sampling was performed by using tubes containing EDTA. Platelet counts and MPV were automatically determined on an ADVIA120 Haematology System (Siemens, Erlangen, Germany) within 1 h after blood sampling. In early foetal demise patients, the MPV and platelet counts were analysed before ultrasound diagnosed as early foetal demise.

### Statistical analysis

All statistical analyses were performed using IBM SPSS Statistics (Version 22.0, SPSS, IBM Corp., Armonk, New York, USA) and R (version 3.4.1, R Foundation for Statistical Computing, Vienna, Austria). Depending on distribution, continuous variables were presented as mean ± Standard Deviation (SD) or median (range), and categorical variables were summarized as counts (frequency percentages). χ^2^ test was applied to compare categorical variables. For the comparison of early foetal demise and pregnant women in the first trimester of pregnancy, Mann–Whitney U test was applied. Then, we attempted to evaluate the association between the risk of early foetal demise and the presence of high platelet counts using binary logistic regression evaluated with the Hosmer-Lemeshow test to accept the models. All non-collinear variables with *p* < .25 in univariable analysis were included in the multivariate regression model. We calculated the odds ratios (ORs) with 95% confidence interval (95% CI) for major risk factors for early foetal demise. Furthermore, the model performance was assessed by 10-fold cross validation by randomly assigning the study subjects and associated data into 10 complementary datasets. Nine datasets were used as the training set and the remaining was used as the validation set. Receiver operator characteristic (ROC) curves (AUCs) from all the testing runs, each point of which corresponds to specific values of true positive rate (sensitivity) and true negative rate (specificity), were averaged and reported. All reported values were two-sided and *p* < .05 was considered as statistical significance.

## Results

### Study population

**Figure 1.**
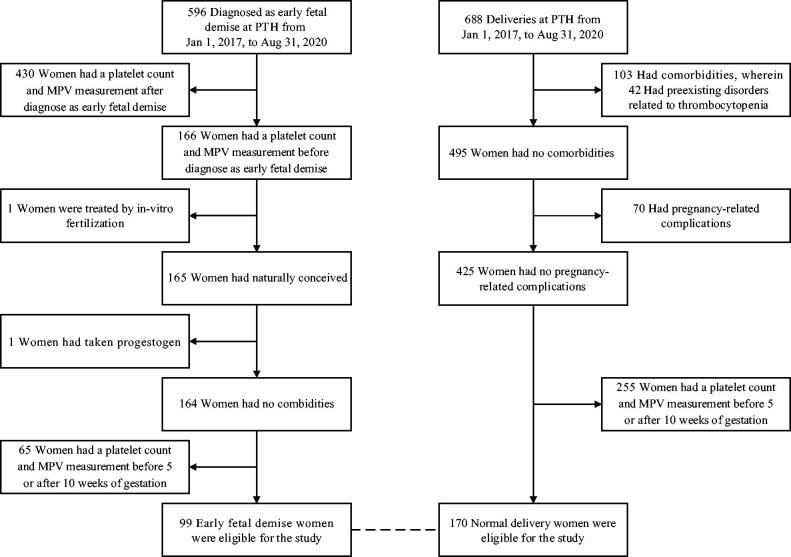
Flow chart of included patients and healthy subjects. Among the 688 deliveries we investigated during our study period, 103 had comorbidities, including 42 having a pre-existing disorder associated with thrombocytopenia, 70 had pregnancy-related complications. One hundred seventy data from the remaining 425 women who had an uncomplicated pregnancy were eligible for our study, and were compared with the data from 99 women identified with early foetal demise. Table 1 shows the baseline characteristics of these women in the normal delivery and early foetal demise groups.

We initially included 688 women who gave birth in the Shanghai Tenth People’s Hospital (TPH) between 1 January 2017 and 31 August 2020 in our study. Among the 688 patients, 103 of them, including 42 with a pre-existing thrombocytopenia, had comorbidities, and were thus excluded from the study. We then excluded 70 of the remaining 495 patients from further analysis due to pregnancy-related complications. One hundred seventy women (median age, 30), whose platelet counts and MPV were measured in the 5–10 weeks of gestation, had an uncomplicated pregnancy and were thus eligible for further analyses (Figure 1). These uncomplicated pregnancies were compared with 99 pregnant women (median age, 32) diagnosed as early foetal demise during the contemporaneous period. The baseline values of the MPV and platelet counts obtained from the women with normal delivery and those diagnosed with early foetal demise are shown in Table 1. We observed high frequency of early foetal demise occurred in the week 8 of gestation.

**Table 1.** Baseline characteristics of participants.

	Normal delivery women (*n* = 170)	Early foetal demise women (*n* = 99)	*p* Value
Age (years)	30 (28–33)	32 (26–38)	.024
BMI (kg/m^2^)^a^	22 (20–24)	21 (19–24)	.598
Parity			
0	113 (66.5%)	36 (36.4%)	<.001
≥ 1	57 (33.5%)	63 (63.6%)	
Miscarriage			
0	101 (59.4%)	37 (37.4%)	<.001
≥ 1	69 (40.6%)	62 (62.6%)	
MPV (fL)	9.25 (8.68–9.90)	9.30 (8.30–10.00)	.802
Platelet counts (×10^9^ per litre)	224 (196–263)	245 (211–278)	.013
VerifyNow Aspirin (ARU)^b^	650 (564–660)	579 (521–623)	.001

Data are median (range) or *n* (%). ^a^Pre-pregnant BMI was missing for 44 patients in the normal delivery group, for 68 patients in the early foetal demise group. ^b^VerifyNow Aspirin was assessed in 23 normal delivery and 50 early foetal demise women.

### Women with early foetal demise

**Figure 2.**
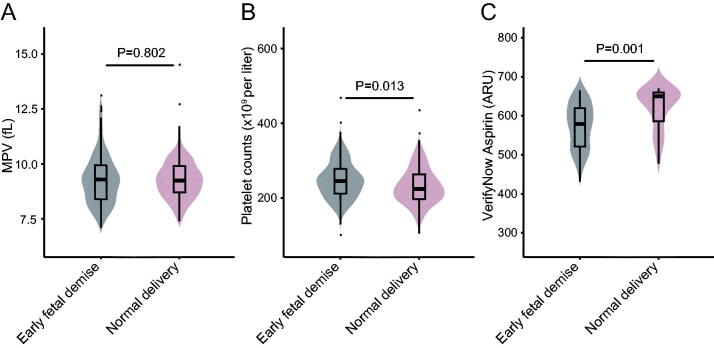
MPV (a), platelet count (b) and VerifyNow Aspirin assay (c) in early foetal demise women, normal delivery women in the 5–10 weeks’ gestation.

We focussed on women with early foetal demise and analysed their platelets in the 5–10 weeks’ gestation before abortion. The median MPV and platelet counts in women with early foetal demise are shown in Table 1. We compared MPV and platelet counts in early foetal demise group with those in the normal delivery group in the 5–10 weeks’ gestation. As shown in Figure 2(a,b), the median platelet counts was 9.4% higher in the early foetal demise group than those in the normal delivery group (245 × 10^9^ vs. 224 × 10^9^ per litre, *p* = .013). However, there was no significant difference of MPV between early foetal demise and normal delivery group (9.30 fL vs. 9.25 fL, *p* = .802). Interestingly, VerifyNow test results demonstrated higher platelet function in the normal delivery group (650 ARU) than the early foetal demise group (579 ARU) (*p* = .001) (Figure 2(c)).

### Risk of early foetal demise in relation to high MPV and platelet counts

**Figure 3.**
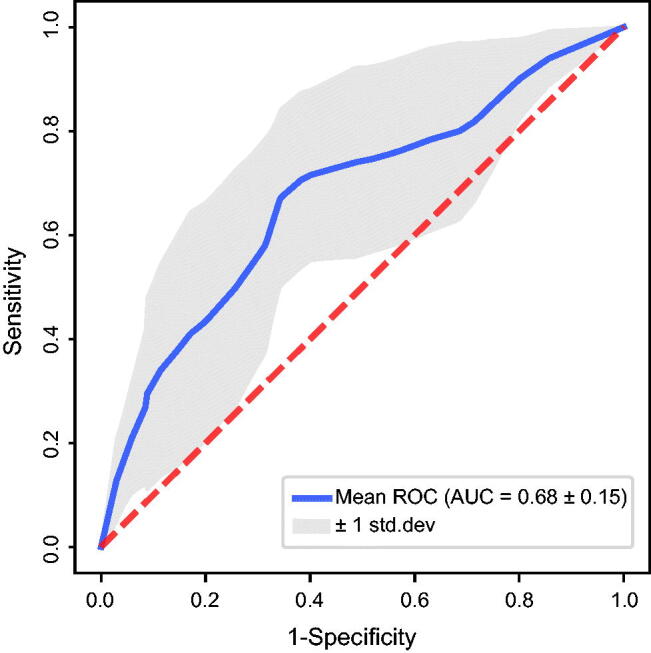
Receiver operating characteristic (ROC) curves for predictors of early foetal demise in 10-fold cross validation. Diagnostic performance of high platelet counts, parity and miscarriage. Area under the curve (AUC) was 0.68 ± 0.15.

We surprisingly found that platelet counts were significantly higher in women with early foetal demise than normal delivery ones in the 5–10 weeks’ gestation. Hence, we attempted to evaluate the association between the risk of early foetal demise and high platelet count levels. All non-collinear significant univariable predictors for early foetal demise were included in the multivariable regression analysis (Table 2). Our results revealed that high platelet count level (>231 × 10^9^ per litre) was independently associated with an increased risk of early foetal demise with an OR of 2.075 (95% CI, 1.215–3.544; *p* = .008). Moreover, in binary logistic regression using forward, backward, and stepwise selection models, high platelet counts, parity and previous miscarriage remained significantly associated with early foetal demise. 10-fold cross validation was performed to assess the overall performance of the model. The average accuracy and precision of 10 folds were 66.59% and 56.80%, respectively. As shown in Figure 3, the average AUC reached 0.68.

**Table 2.** Risk of early foetal demise in relation to platelet counts.

	Cases, *n* (%)		OR (95% CI)	*p* Value
	Normal delivery (*n* = 170)	Early foetal demiss (*n* = 99)		
Univariate analysis				
Age (years)	170 (100.0)	99 (100.0)	1.076 (1.022–1.132)	.005
BMI (kg/m^2^)^a^	127 (74.7)	31 (31.3)	0.985 (0.843–1.151)	.849
Parity				
0	113 (66.5)	36 (36.4)	3.469 (2.065–5.828)	<.001
≥ 1	57 (33.5)	63 (63.6)		
Miscarriage				
0	101 (59.4)	37 (37.4)	2.453 (1.474–4.082)	.001
≥ 1	69 (40.6)	62 (62.6)		
MPV (fL)				
< 9.30	85 (50.0)	47 (47.5)	1.106 (0.674–1.817)	.690
≥ 9.30	85 (50.0)	52 (52.5)		
Platelet counts (×10^9^ per litre)				
< 231.00	96 (56.5)	38 (38.4)	2.083 (1.256–3.454)	.004
≥ 231.00	74 (43.5)	61 (61.6)		
Multivariate analysis				
Age (years)	170 (100.0)	99 (100.0)	0.995 (0.936–1.059)	.881
Parity				
0	113 (66.5)	36 (36.4)	3.002 (1.623–5.552)	<.001
≥ 1	57 (33.5)	63 (63.6)		
Miscarriage				
0	101 (59.4)	37 (37.4)	1.885 (1.077–3.298)	.026
≥ 1	69 (40.6)	62 (62.6)		
Platelet counts (×10^9^ per litre)				
< 231.00	96 (56.5)	38 (38.4)	2.075 (1.215–3.544)	.008
≥ 231.00	74 (43.5)	61 (61.6)		
Overall percentage: 68.8%				

ORs were estimated by binary logistic regression. Ref indicates reference. ^a^Pre-pregnant BMI was missing for 44 patients in the normal delivery group, for 68 patients in the early foetal demise group.

## Discussion

The efficiency of human reproduction maximized at approximately 30% per cycle [23], yet a significant number of these pregnancies end with spontaneous miscarriage. Around one million of early pregnancy loss occur before 12 weeks’ gestation each year in the United States [17,24]. In addition, pregnancy loss before missed menses occurs in a significant proportion of women. The prevalence of early foetal demise was reported around 10% in the UK [25]. Therefore, early prediction of the risk of early foetal demise will improve the efficiency of human reproduction. Currently, there is no reliable biomarker or index to predict the occurrence of early foetal demise. Here we report that high level platelet counts after missed menses is correlated with the risk of early foetal demise.

We observed that platelet counts decreased significantly throughout pregnancy in the normal delivery group, which is consistent with recent report [18]. Additionally, we found that MPV increased significantly from the first trimester of pregnancy in the normal delivery group. However, what the normal physiological range of MPV level during the first trimester of gestation remains unclear. Both genetic and nongenetic factors may influence the level of MPV. Sex, age and white blood cell counts have recently been shown to be the major determinants of MPV heterogeneity [26].

MPV and platelet counts are inversely correlated and maintained homeostasis in healthy individuals [12,13,27,28]. Interestingly, platelet counts in early foetal demise group are higher than those in normal delivery group. Compared with platelet counts in week 5–10 of gestation in normal delivery women, high levels of platelet counts increased the risk of early foetal demise.

A foetal heartbeat may be detectable by a vaginal ultrasound as early as 5 1/2 to 6 weeks’ gestation. After a positive pregnancy test, an early pregnancy ultrasound scan can be performed around 7 to 8 weeks’ pregnancy. In the present study, we focussed on ‘clinical’ pregnancy (>5 weeks after the last menstrual period). We only included patients with 5 to 10 weeks’ pregnancy and diagnosed as early foetal demise. In the present data, we found that high frequency of early foetal demise occurred in the week 8 of gestation, which is consistent with the previous report [29]. We also identified that risk of early foetal demise is in relation to maternal age and pregnancy history, which is in agreement with a recent prospective study [30].

The case-control study design is prone to bias, and we are unable to adjust for risk factors other than age, parity, previous miscarriage in the binary logistic regression model. Furthermore, the association of platelet counts with early foetal demise does not necessarily imply a causal relationship. However, we reasoned that higher levels of platelet counts conferred platelet abnormality and dysfunction in the early stage of pregnancy, which may contribute to early foetal demise. To determine whether the observed increase in platelet counts is cause or consequence of early foetal demise, large prospective studies evaluating platelet counts and platelet function assay before week 10 of gestation in women are needed.

The present study is to examine the risk of early foetal demise conferred by platelet counts in women with pregnancy. The MPV value was recently reported significantly lower in early foetal demise compared to health control [31]. Although there was no difference in MPV between early foetal demise and normal delivery in our study, we expect to validate such an association with prospective studies in future. Additionally, it has been reported that one woman with essential thrombocythaemia developed massive intra-abdominal bleeding after transvaginal oocyte retrieval for *in vitro* fertilization (IVF) [32]. Therefore, efforts should be made to assess the platelet function before IVF. Early monitoring and management of all related risk factors could be immediately implemented and have a positive effect because patients with high platelet counts can easily be identified during routine haematologic analysis.

## Data Availability

The data underlying this article will be shared on reasonable request to the corresponding author.
